# Optimising courier specimen collection time improves patient access to HIV viral load testing in South Africa

**DOI:** 10.4102/ajlm.v11i1.1725

**Published:** 2022-10-25

**Authors:** Sarah J. Girdwood, Thomas Crompton, Naseem Cassim, Floyd Olsen, Portia Sejake, Karidia Diallo, Leigh Berrie, Dorman Chimhamhiwa, Wendy Stevens, Brooke Nichols

**Affiliations:** 1 Health Economics and Epidemiology Research Office, Department of Internal Medicine, School of Clinical Medicine, Faculty of Health Sciences, University of the Witwatersrand, Johannesburg, South Africa; 2 Department of Medical Microbiology, Amsterdam University Medical Center, Amsterdam, the Netherlands; 3 Right to Care, Johannesburg, South Africa; 4 Department of Molecular Medicine and Haematology, Faculty of Health Sciences, University of the Witwatersrand, Johannesburg, South Africa; 5 National Health Laboratory Service, Johannesburg, South Africa; 6 Centers for Disease Control and Prevention, Pretoria, South Africa; 7 Department of Global Health and Development, School of Public Health, Boston University, Boston, Massachusetts, United States

**Keywords:** HIV viral load, scale-up, patient access, South Africa, specimen transport

## Abstract

**Background:**

South Africa uses a courier network for transporting specimens to public laboratories. After the daily collection of specimens from the facility by the courier, patients not yet attended to are unlikely to receive same-day blood draws, potentially inhibiting access to viral load (VL) testing for HIV patients.

**Objective:**

We aimed to design an optimised courier network and assess whether this improves VL testing access.

**Methods:**

We optimised the specimen transport network in South Africa for 4046 facilities (November 2019). For facilities with current specimen transport times (*n* = 356), we assessed the relationship between specimen transport time and VL testing access (number of annual VL tests per antiretroviral treatment patient) using regression analysis. We compared our optimised transport times with courier collection times to determine the change in access to same-day blood draws.

**Results:**

The number of annual VL tests per antiretroviral treatment patient (1.14, standard deviation: 0.02) was higher at facilities that had courier collection after 13:36 (the average latest collection time) than those that had their last collection before 13:36 (1.06, standard deviation: 0.03), even when adjusted for facility size. Through network optimisation, the average time for specimen transport was delayed to 14:35, resulting in a 6% – 13% increase in patient access to blood draws.

**Conclusion:**

Viral load testing access depends on the time of courier collection at healthcare facilities. Simple solutions are frequently overlooked in the quest to improve healthcare. We demonstrate how simply changing specimen transportation timing could markedly improve access to VL testing.

## Introduction

The National Health Laboratory Service (NHLS) is the largest diagnostic pathology service provider in South Africa, providing laboratory and related public health services to over 80% of the population.^1^ Through a national network of laboratories, the NHLS is responsible for supporting the national and provincial health departments in the delivery of healthcare and manages a courier network for transporting specimens from healthcare facilities to centralised testing laboratories. Couriers collect specimens from all health facilities at least once per day and more frequently at high-volume facilities. However, anecdotal evidence suggests that after specimens have been collected by the courier from primary healthcare (PHC) facilities for the day, patients that are still in the queue are unlikely to have their blood drawn that day, potentially inhibiting access to essential diagnostics.

In the case of HIV patients specifically, if the patient is unable to return the following day, viral load (VL) testing could be delayed until the next clinical visit or when the patient is required to collect their next antiretroviral treatment (ART) medication – potentially 2–6 months later – due to inadequate cold-storage facilities at PHC facilities for blood samples. Viral load testing is the recommended method for monitoring HIV patients on ART. South Africa, through the NHLS, currently operates a highly centralised national network that conducted more than five million VL tests at 16 laboratories in 2018.^2^ Despite this, currently, 16% – 31% of HIV-positive South Africans have not had a VL test done within the guideline-recommended window (six months after ART initiation and at 12-month intervals thereafter).^3,4^ Patients with unsuppressed VL for extended periods without clinical action are at risk of increased morbidity, mortality, and/or onward transmission of HIV.^5^ Improving access to VL testing would not only enhance patient-centred care but could result in higher levels of viral suppression.^6^

We aimed to design an optimised courier network that could provide expanded service access to all diagnostics available at PHC facilities within the costs of the current system. We then aimed to assess the impact of the optimised courier network on VL testing access.

## Methods

### Ethical considerations

This project was approved by the University of the Witwatersrand Human Research Ethics Committee (Medical) (HREC) as an additional project within the Integrated Laboratory Data Analysis for Care programme (study approval number: HREC M160978). Additional ethics approval was received from the Center for Global Health, United States Centers for Disease Control and Prevention in Atlanta (study approval number: CGH 2019-224). As this study involved the retrospective analyses of de-identified, aggregated data collected as part of routine care, no patient consent was sought. Although sensitive information was not collected, all data extracted from the NHLS were anonymised, aggregated by facility, and transferred to a secure server. Patients or the public were not involved in the design, conduct, reporting, or dissemination plans of this research.

### Study design

We extended a previously described geospatial cost model to optimise the specimen transport network in South Africa.^7^ Courier routing was optimised for 4046 public facilities in South Africa to improve turnaround times of specimen transport from the health facility to the laboratory. Using data for a sample of PHC facilities for which we had current sample transportation times (*n* = 356), we then used regression analysis to assess the relationship between current specimen transport time and VL testing access. Finally, we compared our specimen transport times from the optimised model with current courier collection times for the subset of facilities (*n* = 356) to determine the percentage change in access to same-day blood draws.

### Network optimisation for healthcare facilities

Data from all public sector healthcare facilities were extracted from NHLS’s Corporate Data Warehouse for a 12-month period (January 2018 to December 2018) and used in the model. Extracted data included the number and type of tests (e.g. VL) requested per site, the location of the site, and the laboratory where specimens were sent to be tested. Data from 4046 healthcare facilities from the NHLS Corporate Data Warehouse were included in the optimisation. These 4046 facilities accounted for 98% of the total test volumes that were couriered during the study period.

Facilities and laboratories in the Corporate Data Warehouse were matched to the District Health Information System so that coordinate data from the District Health Information System could be used to locate the facilities and laboratories. Data quality assessments were conducted on the coordinates to ensure accuracy, and missing coordinate data were sourced from internal implementing partner sources, as well as from Google searches.

The optimised system enhanced the functioning of the current specimen routing network by incorporating factors that would improve specimen validity and would be better aligned with patient-centred care (online Supplementary Text 1).^7^ The cost of the current system was calculated using NHLS expenditure on contracted couriers. The contracted courier rate per kilometre was applied to the distance outputted by the routing optimisation model to estimate the cost of the optimised system.

The optimisation of the specimen transport system was conducted using the ArcGIS Pro Network Analyst tool (Environmental Systems Research Institute, Redlands, California, United States) for vehicle routing problems, which optimises a set of transport routes taking into account the expected specimen volumes, distance from the laboratory to the facility, drive times, and other key constraints identified by stakeholders (online Supplementary Text 2).^8^ A 2019 TomTom (Environmental Systems Research Institute, Redlands, California, United States) routable street data set was sourced from Environmental Systems Research Institute for use in the routing analyses. Relevant outputs from the optimisation included facility name, corresponding route name, time of arrival at the facility by the courier, and the number of VL tests requested by that PHC facility. Using this output, it was possible to determine the average latest time that a courier would collect specimens at a facility per day.

### Change in viral load access for a subset of facilities

To assess whether the optimised specimen transport network improved VL access, we first determined whether facilities that historically had earlier courier collection times had lower VL test volumes compared to facilities with later courier collection times. We then quantified the additional number of patients that would be expected to access VL testing due to the expanded access brought about by delayed courier specimen collection times.

To determine the current latest time at which a health facility is serviced by the courier network, we obtained data on current courier routes and the expected time of arrival at each facility by the courier. Unfortunately, arrival time was not well documented and was only available for a subset of facilities in two provinces, Gauteng and Western Cape (*N* = 497). From this sample, correctional service facilities and hospitals (*N* = 57), as well as facilities that did not have any patients on ART or could not be linked to the District Health Information System (*N* = 80), were removed. Data from the District Health Information System were used to determine the number of patients on ART at the remaining 360 facilities (as of December 2018). Finally, to determine the number of annual VL tests requested by each facility in 2018, the 360 facilities were matched to the NHLS data from the Corporate Data Warehouse. Four facilities were not matched, resulting in a total sample of 356 PHC facilities.

### Data analysis

Using STATA software (version 15.0, College Station, Texas, United States), we performed an unadjusted regression analysis to assess the relationship between current specimen transport time at the 356 PHC facilities and VL testing access, defined as the number of annual VL tests performed per patient on ART.^9^ The independent variable in the regression used the current average specimen transport time as a cut-off, with facilities categorised based on whether their average latest specimen transport time was before or after this cut-off. We also repeated the regression analysis, adjusting for the size of the PHC facility in terms of the number of patients on ART.^10^ A statistical significance cut-off of 5% was used.

We estimated the time distribution of blood draws for patients at PHC facilities based on data from an unpublished time-in-motion study on wait times conducted at an HIV outpatient facility in Johannesburg in 2014.^11^ Using this distribution, it was possible to estimate the number of additional patients that would be able to access a blood draw at different times in the day, depending on the arrival time of the courier.

### Sensitivity

A sensitivity analysis assessed the impact of changing the average latest courier time of the current system on the relationship between the time of the last courier collection for the day and the number of VL tests requested per patient on ART. For each time point (representing the average latest time of courier pick-up at a facility), we determined whether there was a statistically significant difference in the average number of VL tests per patient on ART between facilities whose last courier collection for the day occurred before that point versus those that had courier pick-ups after that point. In addition, we assessed how a different assumption regarding the distribution of patient flow (a more uniform distribution) at a PHC facility would change our results in terms of patient access to same-day blood draws.

## Results

### Network optimisation

The optimised network included 1179 courier routes, involved 136 000 km of driving per day, and serviced the 4046 facilities that are part of the NHLS national network at least once per day. The cost to operate the current system is estimated to be $283 488 United States dollars (USD) per week, and the optimised system costs 1.3% less at $279 901 USD per week. Through specimen network optimisation, the average latest time that patients can access blood draw for laboratory services was delayed to 14:48 from 14:15. In addition, with optimisation, an estimated 85% of specimens arrive at the laboratory by 16:00.

### Impact of optimised network on viral load access

In our sample of facilities for which we had complete current specimen transport times, the weighted average latest time an individual can access a blood draw (weighted by the VL volumes at the facility) is currently 13:36. Under half (141/356; 40%) of the PHC facilities had an average latest courier pick-up time before 13:36, while the average latest courier pick-up time at 215/356 (60%) facilities was after 13:36.

The average number of annual VL tests conducted per patient on ART was higher at facilities with courier pick-up after 13:36 (1.14; standard deviation: 0.02) compared to those with courier pick-up before 13:36 (1.06; standard deviation: 0.03). The number of VL tests conducted per patient on ART was higher (by approximately 0.07) among facilities with courier collection after 13:36 compared to facilities with courier collection before 13:36, and the regression coefficient was significant (*p* < 5%), even when adjusted for PHC facility size (Table 1).

**TABLE 1:** Relationship between average latest courier specimen collection time and average viral load tests per antiretroviral treatment patient† at public sector healthcare facilities in South Africa, January 2018 to December 2018.

Variables	Before 13:36	After 13:36
**Summary statistics**		
Number of facilities	141	215
Average VL tests/ART patient	1.06	1.14
Standard deviation	0.03	0.02
**Regression – dependent variable: ratio of VL tests conducted/ART patient**		
Coefficient	0.08‡	0.07§
Standard error	0.03‡	0.03§
*p*	0.005‡	0.016§

VL, viral load; ART, antiretroviral treatment.

† , Average VL tests/ART patient refers to the average number of VL tests conducted per patient on ART;

‡ , Unadjusted (*n* = 356);

§ , Adjusted (*n* = 356).

**FIGURE 1:**
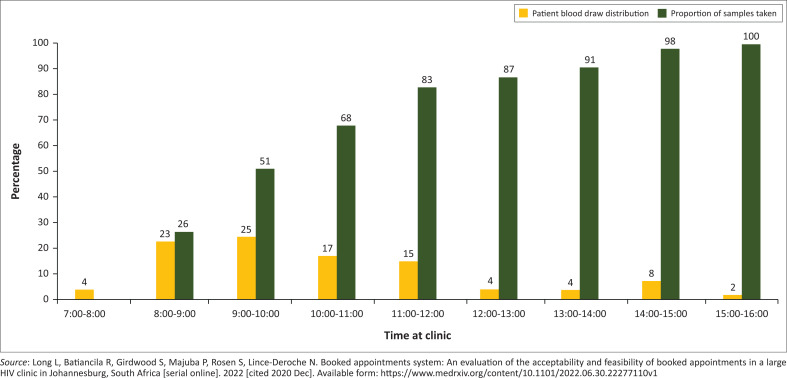
Hourly distribution of patient blood draws at an outpatient clinic, Johannesburg, South Africa, 2014.

Data from an unpublished time-and-motion study on wait times show that the timing of blood draws was skewed to the right, with only 17% of patients getting a blood draw after 12:00 (Figure 1).^11^ The median time for a blood draw was 09:42 (confidence interval: 08:50–11:15) and, on average, patients waited just under 3.5 h from the time that they arrived at the PHC facility until a blood draw was conducted. For our sample of facilities in Gauteng and the Western Cape, we compared the current average latest time for specimen pick-up of 13:36 to the optimised model transport time of 14:35. Based on the distribution of the timing of blood draws, we estimated a 6% increase in patient access to same-day blood draws. With the assumption of a uniform patient distribution over time (i.e. a constant flow of patients for each hour that a health facility is open), the estimated patient access to same-day blood draw increased from 6% to 13%.

**FIGURE 2:**
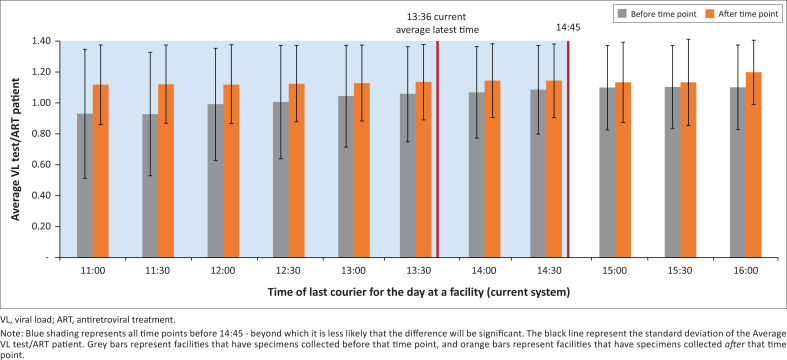
Relationship between time of courier collection and viral load access at public sector healthcare facilities in Gauteng and Western Cape provinces, South Africa, December 2019.

In a sensitivity analysis, facilities that are serviced by a courier earlier in the day are more likely to have a significantly lower number of VL tests conducted per patient on ART (Figure 2). This was true until approximately 14:45, beyond which it was less likely that there would be a significant difference between the number of VL tests conducted per patient on ART at the facilities.

## Discussion

Through the optimisation of the specimen transportation network, the estimated average time of specimen collection was delayed from 14:15 to 14:48 for the entire network of public facilities (*n* = 4046), and from 13:36 to 14:35 for the subset of facilities for which we had complete data (*n* = 356). This is an improvement over the current system as it alleviates the constraint faced by patients and facility staff to quickly collect blood samples before the courier arrives for the final time daily. Earlier specimen arrival enables laboratory staff to meet the daily processing demands as laboratory spikes and workflow could be smoothed out and specimens processed as quickly as possible. Based on our findings, delaying courier collection by an hour can increase patient access to same-day blood draws by 6% – 13% across the Gauteng and Western Cape provinces, depending on the patient flow distribution at the PHC facilities. This simple supply-side intervention to improve the logistics around specimen collection could improve access to VL testing and, consequently, increase VL test volumes and reduce the cost of specimen transportation.

Improving VL testing access in accordance with national guidelines is critical to attaining South Africa’s goal of high levels of viral suppression among HIV patients to meet the last 95% of the HIV targets of the Joint United Nations Programme on HIV and AIDS.^12^ One study conducted in four provinces in South Africa found that only 69% of patients who were due for a VL test had a VL recorded in an electronic register, and only 24% of those who did not have a VL recorded had a blood draw recorded in their file.^3^ This suggests that a lack of blood draws, and not necessarily a failure of the result making it back to the PHC facility, could be a major contributor to the lack of VL results. Furthermore, another study^5^ observed a large number of missing VL test results for patients with a previously unsuppressed VL – patients at greatest risk for HIV morbidity, mortality and onward transmission.^5^ In another study conducted in the Western Cape, 84% had a VL test done when it was due.^4^

Optimising the transportation for VL testing is only one mechanism to improve patient access to VL testing. There are alternative strategies that allow for blood to be drawn at any time of the day. If dried specimens (e.g. dried blood spots or dried plasma spots) were used for VL testing, for which they currently are not, specimens could be taken at any time during the day, even after the courier has picked up the specimens for the last time that day.^13^ Further, should specimen stability be maintained beyond the current recommended limits,^14^ and should guidelines be adjusted to reflect this, this would also be an effective mechanism to increase access to VL testing. Point-of-care tests could also be used, when it is cost-effective, to perform VL testing on demand.^15,16^ Furthermore, where space and resources allow, additional cold-storage facilities could be made available at facilities where current cold storage is inadequate so that after the last courier collection for the day, blood samples can be collected and refrigerated until the next day.

To our knowledge, this is the first paper to model the relationship between specimen transport collection times and VL access in a national VL programme. This analysis relies on innovative geospatial routing optimisation and primary data on laboratory and facility location matched to programmatic ART data.^17^ Although this paper has focused on the impact on VL access, these results are generalisable to other diagnostics where same-day transport is required for specimen integrity.

### Limitations

This study has several limitations. First, we sampled a subset of facilities in only two, predominately urban, provinces out of the nine provinces in South Africa. Nevertheless, while a more representative sample set may have produced different results, this would primarily have been driven by the current average latest time of specimen collection by couriers, which we varied in the sensitivity analysis. The relationship between specimen transport time and VL access would only become insignificant if the average latest time of specimen transport was later than 14:45, which is unlikely to be the case across the network.

Second, this project was conducted in South Africa with an extensive well-functioning specimen referral network that ensures each facility is serviced at least once per day.^18^ Extrapolation of these findings to other countries would require a consideration of the extent of their specimen referral networks. However, for less-developed specimen referral networks, the impact of optimising specimen collection or increasing the frequency of transport such that each facility is serviced at least once a day might result in larger patient access benefits.

Third, we relied on the patient flow distribution at one PHC facility, where the median time of blood draw was 09:42 in the morning, to quantify the patient benefits of increased access. However, for PHC facilities with later median times of blood draw (e.g., with a more uniform distribution of patients throughout the day), the benefits in terms of increased patient access of delaying the courier pick-up time would be greater.

Fourth, we did not quantify the patient benefits of receiving same-day blood draws. Between 6% and 13% of patients requiring a blood draw for VL testing would not need to return to the PHC facility another day, potentially reducing transport costs/opportunity costs of a missed day of work. Patient scheduling and flow may also improve with later specimen transport as facility staff will be aware that there is more time to send patients to the blood draw queue and can thus book patients in for later times in the day, ultimately reducing the waiting time for patients.

Finally, while this project has primarily addressed PHC facilities that predominantly operate during the week and close at 16:00, future research could look at the value of couriers collecting specimens after hours and on weekends at facilities that operate 24 h and/or on weekends. The benefits in terms of increased patient access to VL testing could even be larger as these facilities could potentially offer phlebotomy services to patients after hours and on weekends, where currently there are none.

## Conclusion

Simple solutions are frequently overlooked in our quest to improve healthcare. Delaying specimen pick-up times at PHC facilities by an hour can improve patient access to VL testing at no additional cost. This has implications beyond VL testing and extends to all laboratory diagnostics, especially for specimens whose integrity can be compromised without refrigeration.
